# Fruit

**Published:** 1889-12-07

**Authors:** 


### Words of Consolation.

### FRUIT.

*(For Reading to the Sick.)*

We are all of us very willing to think of the duty of the rich to the poor, of the master to the servant, of God to man, and at once complain if they do not each and all act thoughtfully, kindly, and considerately towards us from our own point of view, leaving out entirely any consideration of the other side of the question. But life is not one-sided. We must look at it all round. For though, unfortunately, sometimes the rich may be mean and stingy, the labour-master tyrannical, unjust, and even cruel, to those in his employ; yet we ought to remember we are the children and servants of One who always acts justly, wisely, and lovingly in all things, and to whom we owe duty and service. Men are constantly in the Bible compared to trees which are expected to bear fruit. If they do so, well and good; if they do not, they are to be cut off and burned. The good man is likened to a tree planted by the rivers of water, which bringeth forth its fruit in due season, and our Blessed Lord tells us that by their fruit men shall be known whether they be good or evil. "Do men," saith He, "gather grapes of thorns or figs of thistles? So by their works ye shall know them."

A very solemn warning is given us in the story of the barren fig tree, which is as follows: "A certain man had a fig tree planted in his vineyard, and he came and sought fruit thereon, and found none. Then said he unto the dresser of his vineyard, Behold these three years I come seeking fruit on this fig tree, and find none. Cut it down; why cumbereth it the ground? And he, answering, said unto him, Lord, let it alone this year also till I shall dig about it, and dung it. And if it bear fruit, well; and if not, then after that thou shalt cut it down." With these words ringing in our ears, let us see how we fail in bringing forth fruit to God's glory. What have we been doing, you and I, with our lives since we were planted by baptism in Christ's vineyard? Have we been trying to do our best to be patient, humble, contented with our lot in life? Are we good tempered to our family, honest, sober, diligent in our work, and serving God with all our hearts? If we can truthfully answer yes, then, indeed, we have been bringing forth our fruit in due season. But if, on the other hand, we cannot say we have made the best of ourselves or our time, if we have professed much and done little at the coming of our Lord, we shall have nothing but a goodly show of leaves. Alas! for us, if He shall say "Cut it down, why cumbereth it the ground?" Year after year He may have watched us and found the same sins, the same failings, the same carelessness about serving Him, the same unloving, ungrateful heart for His blessings. He sees that we require a more severe treatment: gentleness and mercy have had no effect. The sun of righteousness has shone upon us, the dew of the Holy Spirit has watered us, but the tree of our life is utterly worthless. To become fruitful it must be dug about by the sharp prongs of sorrow, it must be pruned by the knife of sickness and distress.

Perhaps you, my good friend, now lying on a bed of sickness, have been such a one, or worse, have loved pleasure in sin and wickedness, without even the green leaves of an outwardly religious life. You have nothing but a barren stump with twisted branches, covered with moss, fit only to be burned. But the merciful Saviour will purge you by His blood, He will lop off the cankered branches, He will make room for the new shoots, and, thus renewed and invigorated, you will be as trees in His vineyard. You must abide in Him and He in you. As a branch cut from a vine withers up and dies, so will you be destroyed if you cast yourself off from His love, His Church, His sacraments. By their help you receive nourishment to grow to His glory. By them you may become saints on earth, whose

Faith is their fixed unswerving root,
Hope their unfading flower,
Fair deeds of charity their fruit,
The glory of their bower.

